# A Shortage of Catgut

**Published:** 1916-06-24

**Authors:** F. C. King

**Affiliations:** Manager. 48 Wigmore Street, Cavendish Square, London, W.


*To the Editor of* The Hospital.

### A Shortage of Catgut.

*To the Editor of* The Hospital.

Sir,—Referring to the extracts given in your issue of June 10 from the London Hospital Report, we notice that mention is made of a shortage of catgut. We should like to draw attention to the fact that for the past three years we have been selling catgut prepared and sterilised by ourselves. Doubtless this information will be of interest to your readers.—We are, Sir, yours faithfully,

For Allen and Hanburys, Ltd.,
F. C. King, Manager.
48 Wigmore Street, Cavendish Square, London, W.,
June 13, 1916.

